# The stage-specific roles of HIF-1α in regulating mESC pluripotency during oxygen transition

**DOI:** 10.1016/j.jbc.2025.110344

**Published:** 2025-06-06

**Authors:** Meng Li, Yang Cao, Huaizhang Jin, Ao Wang, Jian Ruan, Shan Lu, Guosheng Lv, Guoping Zhu, Yang Lei, Xiaopeng Shen

**Affiliations:** 1 College of Life Sciences, Anhui Normal University, Wuhu, Anhui, China; 2 Center for Genetic Medicine, the Fourth Affiliated Hospital of School of Medicine, and International School of Medicine, International Institutes of Medicine, Zhejiang University, Yiwu, China; 3 College of Physical Education, Anhui Normal University, Wuhu, Anhui, China; 4 Center of Reproductive Medicine, Yijishan Hospital of Wannan Medical College, Wuhu, Anhui, China; 5 Wuhu Center for Disease Control and Prevention, Wuhu, Anhui, China

**Keywords:** dynamic regulation, embryonic stem cells, HIF-1α, normoxia–hypoxia transition, pluripotency

## Abstract

Hypoxia-inducible factor 1-alpha (HIF-1α) is a key transcription factor in cellular responses to oxygen levels. This study investigated HIF-1α′s binding dynamics to the genome during the transition from normoxia to hypoxia in mouse embryonic stem cells. Analyzing HIF-1α chromatin immunoprecipitation sequencing data under normoxia, acute hypoxia, and stable hypoxia revealed a "bind–release–bind" pattern, with the weakest binding during acute hypoxia and the strongest during stable hypoxia. Gene Ontology and Kyoto Encyclopedia of Genes and Genomes analyses identified distinct gene sets and pathways regulated by HIF-1α in these conditions, with significant effects on pluripotency under normoxia and stable hypoxia. HIF-1α also partnered with different transcription factors depending on the oxygen level, further influencing its functions. RNA-Seq data and knockdown experiments confirmed the essential role of HIF-1α in maintaining mouse embryonic stem cell pluripotency under normoxia and stable hypoxia, with minimal impact under acute hypoxia. These findings enhance our understanding the regulatory mechanisms of HIF-1α and its role in cellular hypoxic responses.

Hypoxia-inducible factor 1-alpha (HIF-1α) is a critical transcription factor (TF) that orchestrates the cellular response to low oxygen conditions (hypoxia). HIF-1α consists of several essential domains: the basic helix–loop–helix, Per–ARNT–Sim, oxygen-dependent degradation, and transactivation domains (1, 2). It plays a pivotal role in regulating genes involved in angiogenesis, metabolism, cell survival, and development (3, 4, 5). Under normoxic conditions, prolyl hydroxylases hydroxylate HIF-1α, promoting its interaction with the von Hippel–Lindau E3 ubiquitin ligase complex, which leads to its degradation. In hypoxic conditions, prolyl hydroxylase activity is inhibited because of lack of oxygen, allowing HIF-1α to accumulate, dimerize with HIF-1β, and activate the transcription of target genes by binding to hypoxia-response elements in their promoters (6). For instance, *Pgk1* and *Ldha* are commonly recognized as HIF-1α target genes (7, 8). However, oxygen concentration broadly affects cellular states. Hypoxia impacts DNA methylation, histone modification, and chromatin reprogramming, as previously reported (9). The differences and functions of HIF-1α target genes under stable normoxia, stable hypoxia, and the transition stage between these two stages remain unclear.

Embryonic stem cells (ESCs) were derived from the inner cell mass of blastocytes and retained the capability to differentiate into all kinds of cells within embryos. ESCs are widely used in the studies of developmental biology, signaling transduction, pharmacology, and others. The *in vitro* culture of ESCs relied on the proper maintenance of ESC pluripotency, which was currently achieved by supplementing pluripotency maintaining reagents, introducing genetic modifications, and providing pluripotency-favorable environmental conditions (10). In particular, it is necessary to add leukemia inhibitory factor (LIF) in mouse ESCs (mESCs) to maintain pluripotency (11, 12), whereas fibroblast growth factor and activin A are required in human ESCs (13, 14). Hypoxia and HIF-1α have previously been reported as key factors affecting the pathways associated with ESC pluripotency, although the results have been controversial. Hypoxia and HIF-1α promote the Wnt/β-catenin signaling pathway in mESCs, which is crucial for maintaining stem cell stemness and pluripotency (15). In addition, HIF-1α can transcriptionally regulate other hypoxia-responsive genes, promoting a metabolic shift toward glycolysis and maintaining the cellular environment necessary for stem cell pluripotency (16). However, other studies argue that HIF-1α downregulates key pluripotency genes and promotes differentiation, particularly toward mesoderm and endoderm lineages (17, 18). Furthermore, the effects of hypoxia on ESCs were highly divergent and largely impacted by oxygen contents and hypoxic treatment timing (19, 20). These conflicting results have limited the application of hypoxia and HIF-1α in manipulating ESC pluripotency status.

In this study, we profiled the transcriptional targets of HIF-1α during the transition from normoxia to hypoxia in mESCs. We found that compared with stable normoxia and hypoxia, HIF-1α regulated the fewest genes at the intermediate transition stage, despite its peak expression at this time. In contrast, HIF-1α regulated pluripotency-related genes, maintaining mESC pluripotency under stable normoxia and hypoxia but not at the transition stage. These results suggested that HIF-1α binds different sets of target genes and exerts distinct biological functions under varying atmospheric conditions.

## Results

### The dynamics of HIF-1**α** as a TF during the transition from normoxia to hypoxia

HIF-1α is well known for its essential role in mediating the response to hypoxia treatment. However, it was recently reported that HIF-1α is also expressed and exerts important functions under normoxia (21, 22). Despite this, it remained unclear whether HIF-1α transcriptionally regulates the same target genes under normoxia and hypoxia and the dynamics of HIF-1α binding to the genome during the transition between the two conditions.

**Figure 1:**
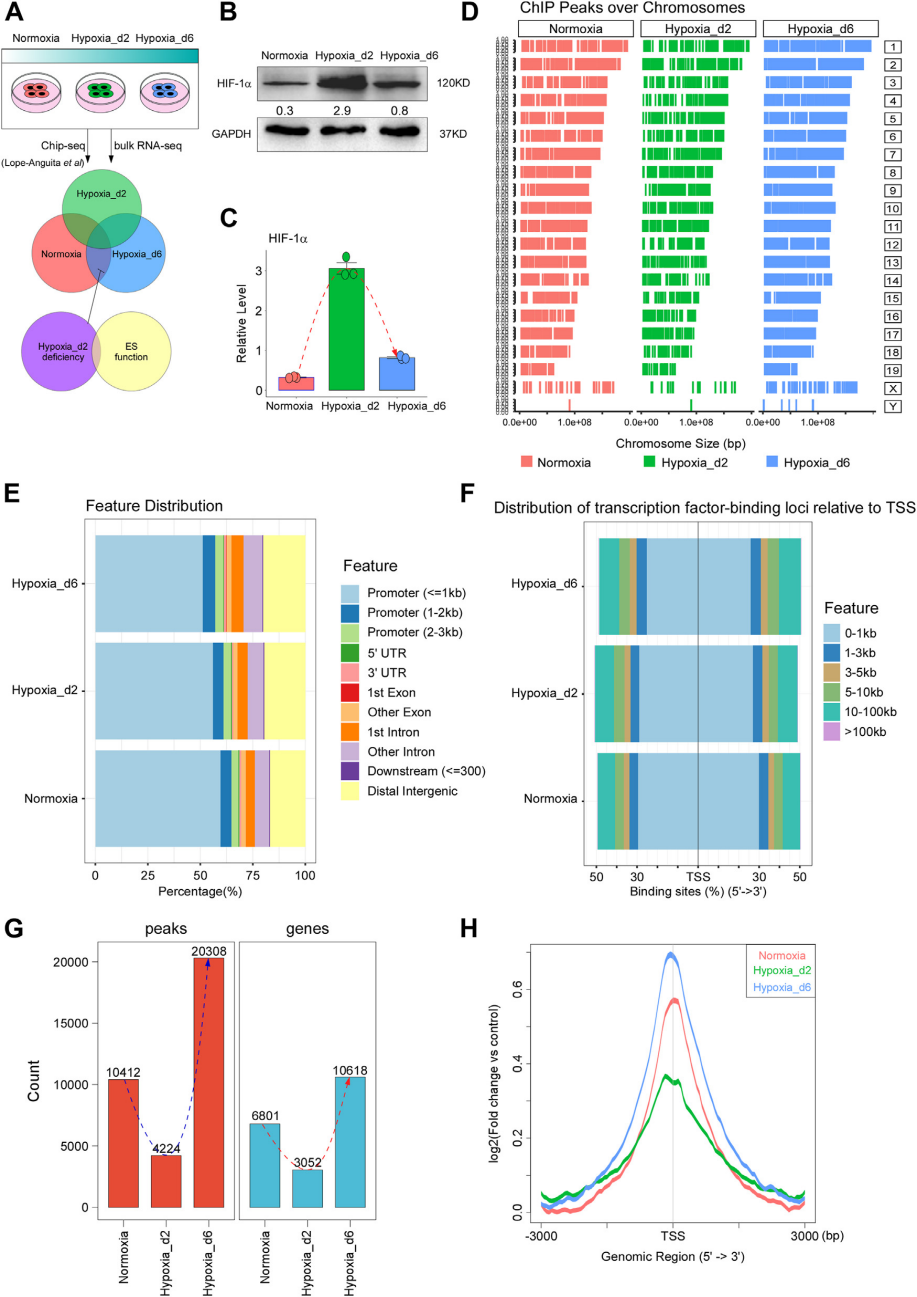
**The dynamics of HIF-1α as a transcription factor during the transition from normoxia to hypoxia.** The HIF-1α ChIP-Seq in the Normoxia, Hypoxia_d2, and Hypoxia_d6 groups was analyzed. *A*, the schematic diagram showing the overall design of this study. *B* and *C*, the expressions of HIF-1α in the three groups were determined by Western blots; independent replications, n = 3. *D*, the overall distributions of HIF-1α peaks on the mouse genome in the three groups. *E*, the distributions of HIF-1α peaks on different gene regions in the three groups. *F*, the overall distances of HIF-1α peaks to the TSS of target genes. *G*, the total peaks and genes that HIF-1α targeted in the three groups. *H*, the overall binding strength of HIF-1α in the three groups. ChIP-Seq, chromatin immunoprecipitation; HIF-1α, hypoxia-inducible factor 1-alpha; TSS, transcription start site.

In this study, we analyzed HIF-1α chromatin immunoprecipitation sequencing (ChIP–Seq) data during the transition from normoxia to hypoxia to depict the genome binding profile of HIF-1α. The data included ChIP-Seq assays against HIF-1α performed on mESCs under three different conditions: 1) mESCs stably cultured under normoxia (termed “Normoxia”); 2) mESCs just switched from normoxia to hypoxia and cultured under hypoxia for only 2 days, resembling the intermediate stage of the transition (termed “Hypoxia_d2”); and 3) mESCs stably cultured under hypoxia for 6 days, resembling the stable hypoxia stage (termed “Hypoxia_d6”) (Fig. 1*A*). To exclude the possibility that HIF-1α was not expressed, we performed Western blots on the mESCs of these three groups. HIF-1α was evidently expressed in all three groups, with its expression in the Hypoxia_d2 group being the highest (Fig. 1, *B* and *C*). We then analyzed the ChIP-Seq data of the three groups and mapped all the identified peaks onto the mouse genome. The overall distribution patterns of the three groups were identical (Fig. 1*D*). Next, we analyzed the location of HIF-1α peaks in the three groups and found that the proportions of HIF-1α binding to promoter regions gradually decreased during the transition from normoxia to hypoxia, whereas the proportions of HIF-1α binding to distal intergenic and intron regions gradually increased (Fig. 1*E*). Meanwhile, we determined the distances of HIF-1α peaks to the transcription start site (TSS) of target genes. The result showed that HIF-1α was prone to bind to sites far from the TSS of target genes as the transition from normoxia to hypoxia (Fig. 1*F*).

Furthermore, we performed statistical analyses on the total peaks and genes that HIF-1α targeted in the three groups (Fig. 1*G*). Surprisingly, the Hypoxia_d2 group displayed the fewest peaks and gene numbers, which is opposite to the HIF-1α expressions in the three groups (Fig. 1, *B* and *C*). Notably, the Hypoxia_d6 group had the most HIF-1α-targeted peaks and genes, agreeing with the established role of HIF-1α under hypoxia.

Finally, we analyzed the overall binding strength of HIF-1α in the three groups and found that the binding of HIF-1α to targets was the highest in the Hypoxia_d6 group and the lowest in the Hypoxia_d2 group, with an intermediate level in the Normoxia group (Fig. 1*H*). These results together implied that during the transition from normoxia to hypoxia, HIF-1α was transiently released from many of its targeted genome sites under normoxia and then rebound to its targeted genome sites under hypoxia. Moreover, the hypobinding of HIF-1α was not because of its expression levels, as HIF-1α exhibited the highest expression at the intermediate stage of the transition from normoxia to hypoxia.

### HIF-1**α** transcriptionally regulates variable biological processes and pathways during the transition from normoxia to hypoxia

As the previous results indicated that HIF-1α exhibited a "bind–release–bind" pattern during the transition from normoxia to hypoxia, we investigated whether HIF-1α binds and regulates different genes, thereby affecting distinct biological processes and pathways under these conditions.

**Figure 2:**
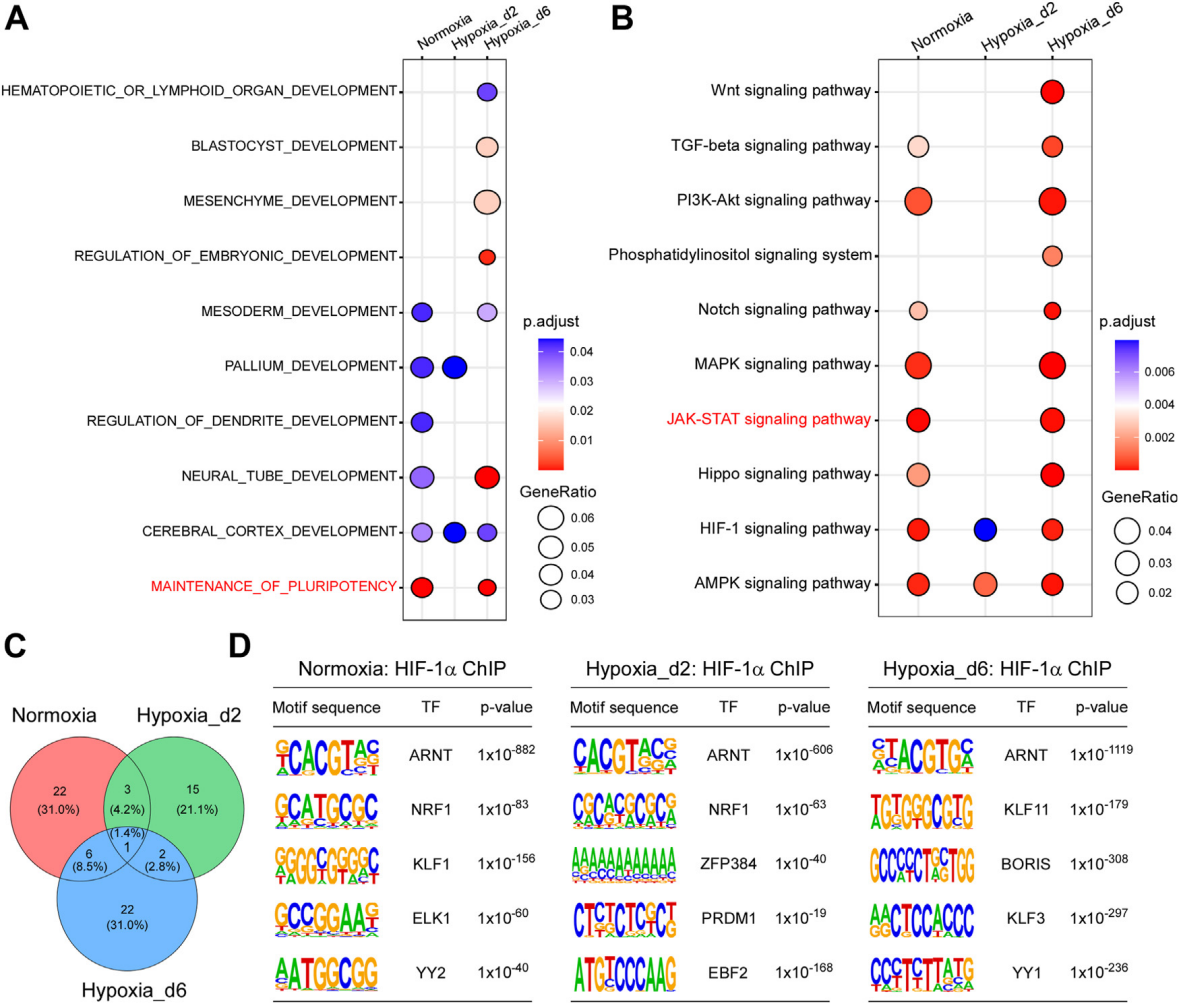
**HIF-1α transcriptionally regulated variable biological processes and pathways during the transition from normoxia to hypoxia.***A*, the Gene Ontology (GO) analysis of development-related processes on the HIF-1α target genes in the three groups. *B*, the KEGG pathway analysis on the target genes of HIF-1α for the three groups. *C*, the Venn plot showing the TFs that HIF1-α might cooperate with in the three groups. *D*, top five enriched TFs of the three groups. HIF-1α, hypoxia-inducible factor 1-alpha; KEGG, Kyoto Encyclopedia of Genes and Genomes; TF, transcription factor.

First, we performed Gene Ontology (GO) analysis of development-related processes on the HIF-1α target genes in the three groups. As a result, in the Hypoxia_d2 group, HIF-1α target genes did not show enrichment in processes related to stem cell pluripotency maintenance, in contrast to the Normoxia and Hypoxia_d6 groups (Fig. 2*A*). In these latter groups, HIF-1α significantly regulated multiple pathways with well-established roles in stem cell function and differentiation, including the Janus kinase–signal transducer and activator of transcription (JAK–STAT) pathway (Fig. 2*B*) Notably, the Hypoxia_d2 group regulated the fewest biological processes and pathways, consistent with the finding that the number of peaks and genes bound by HIF-1α was the least (Fig. 1*G*). These results suggested that the dynamics of HIF-1α during the transition between normoxia and hypoxia mediated biased cellular processes and pathways, potentially orchestrating different fates of mESCs.

To explore the TFs that HIF-1α might cooperate with in the three groups, we performed motif analysis on the HIF-1α binding peaks for each group. We identified 32, 20, and 31 predicted TFs for Normoxia, Hypoxia_d2, and Hypoxia_d6, respectively, based on the consensus binding motifs of TFs. The HIF-1α and ARNT complex was the only TF shared across the three groups. The Hypoxia_d2 group shared four TFs with the Normoxia group and three TFs with the Hypoxia_d6 group; the Normoxia and Hypoxia_d6 groups shared seven TFs (Fig. 2*C*). The top enriched TFs for each group were largely distinct (Fig. 2*D*). These differences in cooperating TFs might partially explain the biased target genes and their enriched biological processes and pathways regulated by HIF-1α in the three groups. Taken together, these results suggested that the genes, biological processes, and pathways regulated by HIF-1α dynamically change during the transition from normoxia to hypoxia.

### Differential gene sets dynamically regulated by HIF-1**α** during the transition from normoxia to hypoxia

**Figure 3:**
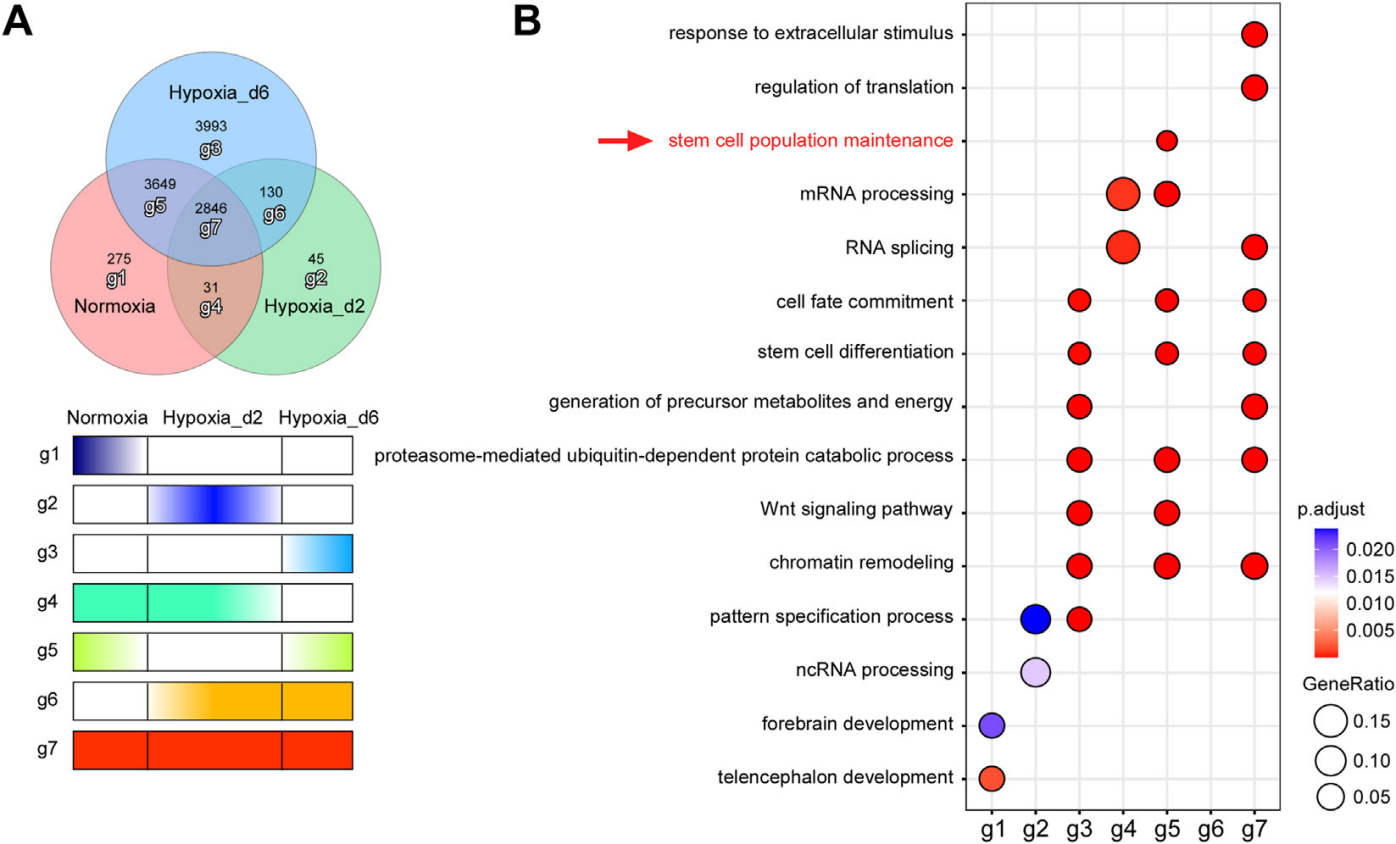
**Differential gene sets dynamically regulated by HIF-1α during the transition from normoxia to hypoxia.***A*, the seven gene sets dynamically targeted by HIF-1α during the transition from normoxia to hypoxia. *B*, the GO analysis on the seven gene sets. GO, Gene Ontology; HIF-1α, hypoxia-inducible factor 1-alpha.

To determine the specific genes regulated by HIF-1α at different stages during the transition from normoxia to hypoxia, we classified all HIF-1α targets from the Normoxia, Hypoxia_d2, and Hypoxia_d6 groups into seven gene sets (Fig. 3*A*). The gene sets g1, g2, and g3 contained group-specific target genes for the Normoxia, Hypoxia_d2, and Hypoxia_d6 groups, respectively. The gene sets g4, g5, and g6 included overlapping target genes of Normoxia and Hypoxia_d2, Normoxia and Hypoxia_d6, and Hypoxia_d2 and Hypoxia_d6, respectively. The g7 gene set comprised genes common to all three groups.

We plotted a heatmap showing the binding strength of HIF-1α to all gene sets in each group (Fig. S1*A*). GO analysis of these gene sets revealed that the g5 gene set, regulated by HIF-1α in a stage-specific manner, and g7 gene set primarily contribute to the positive regulation of cell fate commitment and stem cell differentiation. Notably, the g5 cluster exhibits a unique and positive regulatory role in maintaining stem cell pluripotency (Fig. 3*B*). Furthermore, the JAK–STAT signaling pathway, which plays a key role in the maintenance of pluripotency, was uniquely enriched in the g5 cluster, highlighting its potential functional importance (Fig. S1*B*). These results imply that HIF-1α may regulate the maintenance of stem cell pluripotency in a stage-specific manner through the JAK–STAT signaling pathway.

**Figure 4:**
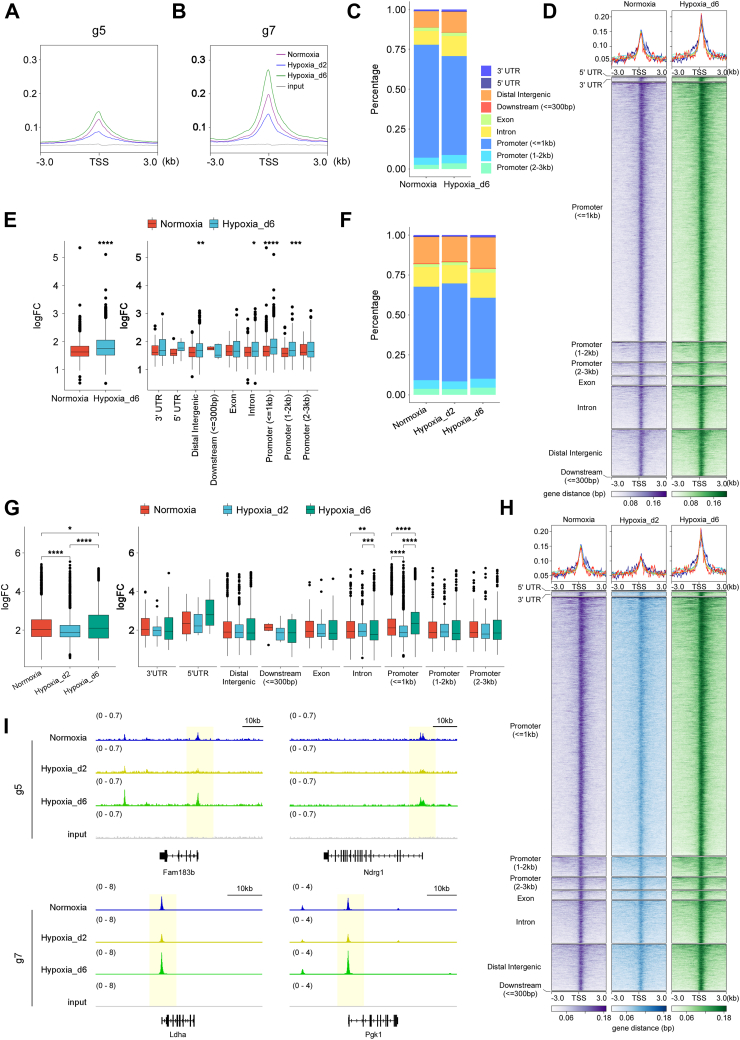
**The comparisons of HIF-1α binding to the g5 and g7 gene sets in Normoxia, Hypoxia_d2, and Hypoxia_d6 groups.** The overall binding strength on the (*A*) g5 and (*B*) g7 genes of HIF-1α in the three groups. *C*–*E*, the gene regions of the g5 genes that HIF-1α targeted in the three groups. *F*–*H*, the gene regions of the g7 genes that HIF-1α targeted in the three groups. *I*, the HIF-1α ChIP-Seq peaks at the loci of *Fam183b*, *Ndrg1*, *Ldha*, and *Pgk1* in the three groups. ∗*p* < 0.05; ∗∗*p* < 0.01; ∗∗∗*p* < 0.001; and ∗∗∗∗*p* < 0.0001. ChIP-Seq, chromatin immunoprecipitation; HIF-1α, hypoxia-inducible factor 1-alpha.

Focusing on the g5 and g7 gene sets, we examined the overall binding strength of HIF-1α in the Normoxia, Hypoxia_d2, and Hypoxia_d6 groups for these genes (Fig. 4, *A* and *B*). The overall binding strength of HIF-1α was higher for g7 genes compared with g5, which may explain the consistent binding and regulation of g7 genes, whereas a "bind–release–bind" pattern was observed for g5 genes during the transition.

We then analyzed the gene regions of g5 and g7 genes targeted by HIF-1α in the three groups. For g5 genes, HIF-1α peaks were more often located at distal intergenic regions and less frequently at promoter (≤1 kb) regions in the Hypoxia_d6 group compared with the Normoxia group (Fig. 4*C*). HIF-1α exhibited significantly enhanced binding to distal intergenic, intron, promoter (≤1 kb), and promoter (1 and 2 kb) regions of g5 genes in the Hypoxia_d6 group *versus* the Normoxia group (Fig. 4, *D* and *E*). For g7 genes, HIF-1α peaks were more enriched at promoter (≤1 kb) regions in the Hypoxia_d2 group compared with the Normoxia and Hypoxia_d6 groups (Fig. 4*F*), but the overall binding strength of HIF-1α was the lowest in these promoter regions in the Hypoxia_d2 group (Fig. 4, *G* and *H*). These results collectively suggested that the binding site and strength of HIF-1α underwent dynamic changes during the transition from normoxia to hypoxia.

We selected two representative genes from the g5 and g7 sets, respectively. *Fam183b* and *Ndrg1*, part of the g5 genes, exhibited HIF-1α binding at their loci in the Normoxia and Hypoxia_d6 groups but scarcely any in the Hypoxia_d2 group. *Ldha* and *Pgk1*, two canonical HIF-1α transcriptional targets in the g7 set, showed HIF-1α binding at their loci across all three groups (Fig. 4*I*). These findings further demonstrated that HIF-1α binding was significantly altered in terms of gene subregions and binding strength under different conditions, affirming that the function of HIF-1α as a TF is highly dynamic.

### Biased binding of HIF-1**α** resulted in differential regulatory roles in mESC pluripotency during the transition from normoxia to hypoxia

The aforementioned results suggested that during the transition from normoxia to hypoxia, HIF-1α displayed a "bind–release–bind" pattern in genome binding, with the lowest number of bound genes and overall binding strength observed in the Hypoxia_d2 group (Fig. 1, *G* and *H*). This intriguing finding led us to explore the specific profiles of mESC gene expressions and HIF-1α functions in the Hypoxia_d2 group.

**Figure 5:**
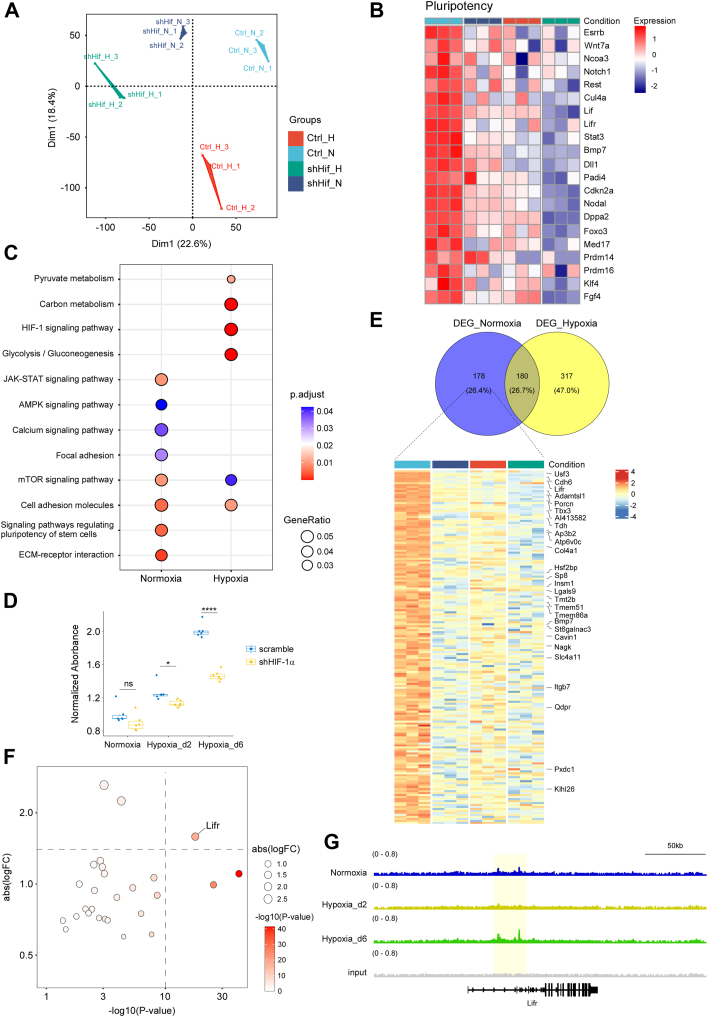
**The biased binding of HIF-1α resulted in its differential regulatory roles in mESC pluripotency during the transition from normoxia to hypoxia.** We performed RNA-Seq on these four groups: (1) scramble control under normoxia (Ctrl_N); (2) HIF-1α knockdown under normoxia (shHif_N); (3) scramble control under hypoxia for 2 days (Ctrl_H); and (4) HIF-1α knockdown under hypoxia for 2 days (shHif_H). *A*, the PCA on the RNA-Seq data of all the four groups. *B*, the heatmap showing the expressions of pluripotency-related genes in the four groups. *C*, the KEGG pathway analysis on the downregulated DEGs from the DEGs of the shHif_N *versus* Ctrl_N and shHif_H *versus* Ctrl_H. *D*, a lactate assay to evaluate glycolytic activity in both scramble control and HIF-1α knockdown mESCs under normoxia, 2-day acute hypoxia (Hypoxia_d2), and 6-day stable hypoxia (Hypoxia_d6). The relative lactate level was measured by absorbance at 530 nm. All values were normalized to the scramble group under normoxia. n = 6. ∗*p* < 0.05; ∗∗∗∗*p* < 0.0001; ns, not significant. *E*, the heatmap showing the 178 remarkably repressed by HIF-1α knockdown under normoxia. *F*, the dotplot showing the log2(fold change) and *p* values of 27 genes that belonged to both the 178 normoxia-specific genes in (*E*) and the g5 gene set. *G*, the HIF-1α ChIP-Seq peaks at the loci of *Lifr* in the Normoxia, Hypoxia_d2, and Hypoxia_d6 groups. ChIP-Seq, chromatin immunoprecipitation sequencing; DEG, differentially expressed gene; HIF-1α, hypoxia-inducible factor 1-alpha; KEGG, Kyoto Encylopedia of Genes and Genomes; mESC, mouse embryonic stem cell; PCA, principal component analysis.

To investigate this, we constructed scramble control and HIF-1α knockdown mESC cell lines. These cell lines were initially cultured under normoxia and then divided into two groups: one continued under normoxia, and the other switched to hypoxia for 2 days. We performed RNA-Seq on four groups: 1) scramble control under normoxia (Ctrl_N); 2) HIF-1α knockdown under normoxia (shHif_N); 3) scramble control under hypoxia for 2 days (Ctrl_H); and 4) HIF-1α knockdown under hypoxia for 2 days (shHif_H). Principal component analysis of the RNA-Seq data showed distinct separation among the four groups (Fig. 5*A*), implying that both hypoxia and HIF-1α significantly affect gene expression in mESCs.

Pluripotency, a key feature of mESCs, was previously found to be regulated by hypoxia and HIF-1α, though results were inconsistent (16, 18). Therefore, we examined the expression of pluripotency-related genes in the four groups. Acute hypoxia treatment for 2 days (Ctrl_H *versus* Ctrl_N) significantly reduced the expression of pluripotency-related genes, indicating hypoxia-induced pluripotency loss, which may facilitate stem cell differentiation. To further support this notion, we performed ChIP-Seq experiments targeting H3K4me3 and H3K27ac in mESCs cultured under normoxia and 2-day hypoxia. These histone marks are widely recognized indicators of active gene transcription. In our newly generated dataset, we observed a significant reduction in H3K4me3 and H3K27ac signals at core pluripotency gene loci (*e.g.*, *Nanog*, *Esrrb*, *Oct4*, *Sox2*) following acute hypoxia treatment (Fig. S2). HIF-1α knockdown under normoxia (shHif_N *versus* Ctrl_N) also significantly downregulated pluripotency-related genes, highlighting the essential role of HIF-1α in maintaining mESC pluripotency under normoxia. Under hypoxia for 2 days, HIF-1α knockdown (shHif_H *versus* Ctrl_H) resulted in slight reductions in pluripotency-related gene expression, though less pronounced than under normoxia (Fig. 5*B*). These findings suggested that HIF-1α had a more significant effect on mESC pluripotency under normoxia than under acute hypoxia.

The g5 gene set, targeted by HIF-1α under stable normoxia and hypoxia but not under acute hypoxia (Fig. 3*A*), was the only gene set regulating stem cell pluripotency and maintenance (Figs. 3*B*, S1*B*). This led us to hypothesize that biased binding of HIF-1α to certain pluripotency genes in the g5 set results in biased effects on mESC pluripotency.

Next, we analyzed gene expression changes because of HIF-1α knockdown under normoxia and acute hypoxia for 2 days. We identified differentially expressed genes (DEGs) in shHif_N *versus* Ctrl_N and shHif_H *versus* Ctrl_H, respectively. Kyoto Encyclopedia of Genes and Genomes (KEGG) pathway analysis of downregulated DEGs showed that mechanistic target of rapamycin and cell adhesion molecule pathways were enriched under both conditions. Under normoxia, pathways like JAK–STAT and pluripotency regulation were significantly altered. In contrast, under hypoxia, pathways related to glycolysis were significantly affected (Fig. 5*C*).

To further investigate glycolytic activity, we performed a lactate assay to evaluate glycolytic activity in both scramble control and HIF-1α knockdown mESCs under three oxygen conditions: normoxia, 2-day acute hypoxia (Hypoxia_d2), and 6-day stable hypoxia (Hypoxia_d6). The results showed that lactate levels rose progressively during the oxygen shift, reflecting heightened glycolytic activity upon hypoxia induction and maintenance. Furthermore, HIF-1α knockdown significantly attenuated lactate accumulation under both acute (Hypoxia_d2) and stable hypoxia (Hypoxia_d6). Under normoxia, baseline lactate levels remained low, and although HIF-1α depletion trended downward, the reduction did not reach statistical significance (Fig. 5*D*). Notably, the pathways regulating stem cell pluripotency and JAK–STAT, significantly enriched in the g5 set, were only regulated by HIF-1α under normoxia (Figs. 3*B*, S1*B*).

Comparing DEGs under normoxia and hypoxia for 2 days revealed 178 normoxia-specific, 317 hypoxia-specific, and 180 shared downregulated DEGs. The 178 normoxia-specific genes were significantly repressed by HIF-1α knockdown under normoxia but not under acute hypoxia (Fig. 5*E*). Among these, 27 genes belonged to the g5 set. Analysis of log_2_(fold change) and *p* values in shHif_N *versus* Ctrl_N identified *Lifr* as the most altered gene (Fig. 5*F*). HIF-1α ChIP-Seq data confirmed that *Lifr* was bound by HIF-1α under normoxia and Hypoxia_d6 but not Hypoxia_d2 (Fig. 5*G*). This suggested that HIF-1α transcriptionally regulated *Lifr* expression, which was disrupted under acute hypoxia because of HIF-1α dissociation from the *Lifr* locus. LIFR, a receptor for LIF and an essential pluripotent factor, activates the JAK–STAT pathway, indicating that dynamic HIF-1α binding affects mESC pluripotency profoundly. These findings highlighted the dynamic and context-dependent role of HIF-1α in regulating mESC pluripotency during the transition from normoxia to hypoxia.

### The role of HIF-1**α** in pluripotency under stable normoxia and hypoxia but not acute hypoxia

**Figure 6:**
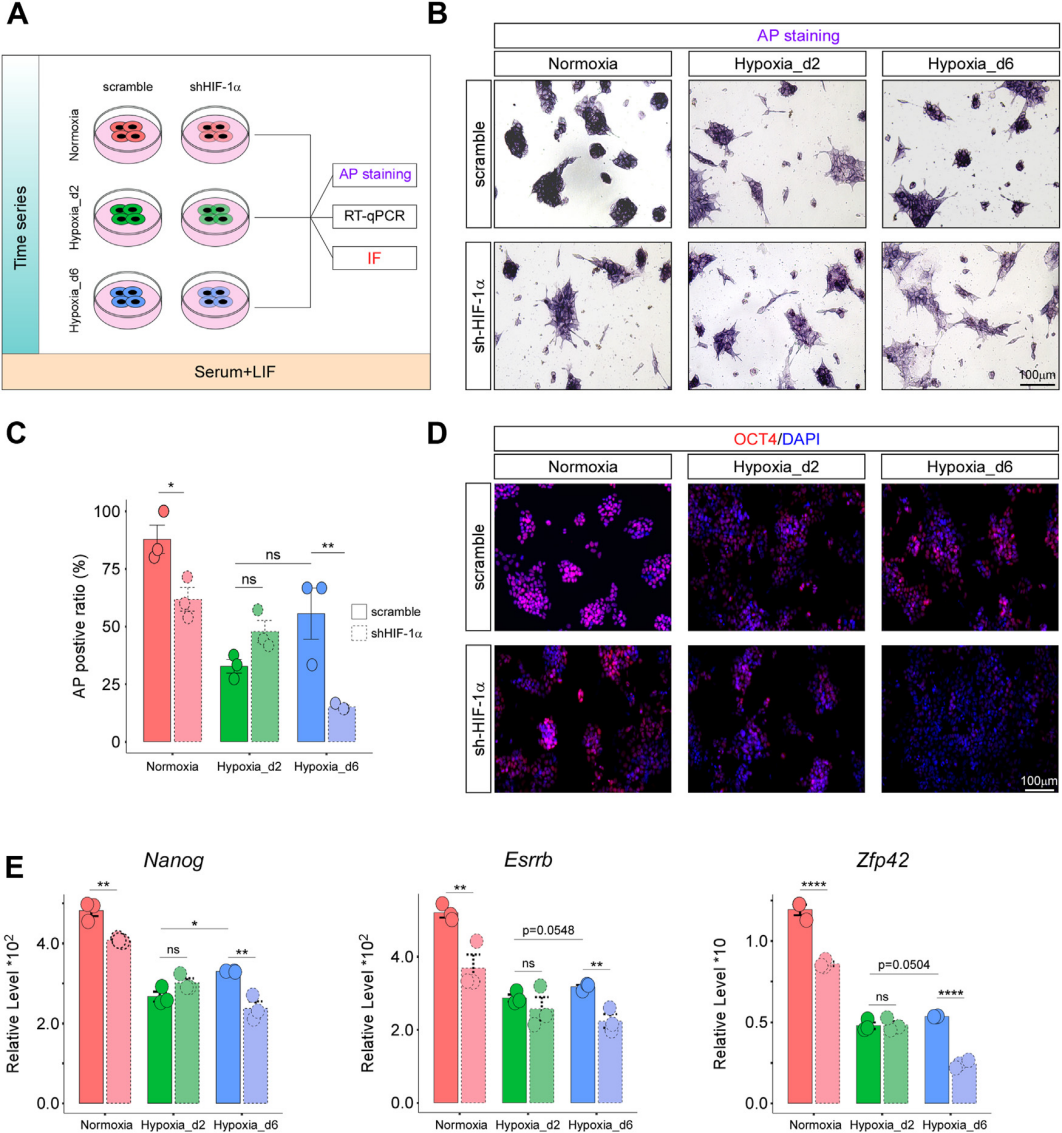
**HIF-1α was responsible for the pluripotency under stable normoxia and hypoxia but not under acute hypoxia.***A*, the schematic diagram showing the pluripotency assays performed on scramble and shHIF-1α mESCs that were cultured normoxia (Normoxia), hypoxia for 2 days (Hypoxia_d2), and hypoxia for 6 days (Hypoxia_d6), respectively. (*B* and *C*), the AP stainings, (*D*) OCT4 immunostaining, and (*E*) RT–quantitative PCR of *Nanog*, *Esrrb*, and *Zfp42* on all assay groups; independent replications, n = 3. ∗*p* < 0.05; ∗∗*p* < 0.01; ∗∗∗∗*p* < 0.0001; ns, not significant. AP, alkaline phosphatase; HIF-1α, hypoxia-inducible factor 1-alpha; mESC, mouse embryonic stem cell.

The results indicated that HIF-1α transcriptionally regulated genes associated with stem cell pluripotency in the Normoxia and Hypoxia_d6 groups but not in the Hypoxia_d2 group (Fig. 3, *C* and *D*). Furthermore, HIF-1α knockdown significantly suppressed stem cell pluripotency under normoxia but not under acute hypoxia for 2 days (Fig. 5, *B* and *C*). To further validate the role of HIF-1α in mESC pluripotency, we conducted a series of pluripotency assays on scramble control (scramble) and HIF-1α knockdown (shHIF-1α) mESCs cultured under normoxia (Normoxia), hypoxia for 2 days (Hypoxia_d2), and hypoxia for 6 days (Hypoxia_d6) (Fig. 6*A*).

During these assays, mESCs were cultured in serum-containing medium supplemented with LIF, a widely used condition that maintains mESC pluripotency (11, 23). As expected, the scramble–Normoxia group maintained high alkaline phosphatase (AP) activities (Fig. 6, *B* and *C*) and high expression levels of pluripotency markers (OCT4, *Nanog*, *Esrrb*, and *Zfp42*) (Fig. 6, *D* and *E*), indicating that wildtype mESCs cultured under these conditions preserved their pluripotency. However, both the scramble–Hypoxia_d2 and scramble–Hypoxia_d6 groups showed reduced AP activities and decreased expression of pluripotency markers compared with the scramble–Normoxia group, suggesting that both acute and chronic hypoxia negatively impacted mESC pluripotency (Fig. 6, *B*–*E*).

Interestingly, shHIF-1α mESCs exhibited significantly reduced AP activities and lower expression of pluripotency markers in the Normoxia and Hypoxia_d6 groups but not in the Hypoxia_d2 group. This finding suggested that HIF-1α regulated mESC pluripotency under stable normoxia and hypoxia, but not under acute hypoxia, aligning with our previous analyses (Figs. 3*B*, S1*B*, 5*C*). Notably, the scramble–Hypoxia_d2, scramble–Hypoxia_d6, and shHIF-1α–Hypoxia_d2 groups showed similar levels of AP activities and pluripotency marker expression, whereas the shHIF-1α–Hypoxia_d6 group exhibited the lowest levels of these indicators. These results implied that HIF-1α played a crucial role in maintaining mESC pluripotency under stable normoxia and hypoxia but not under acute hypoxia.

**Figure 7:**
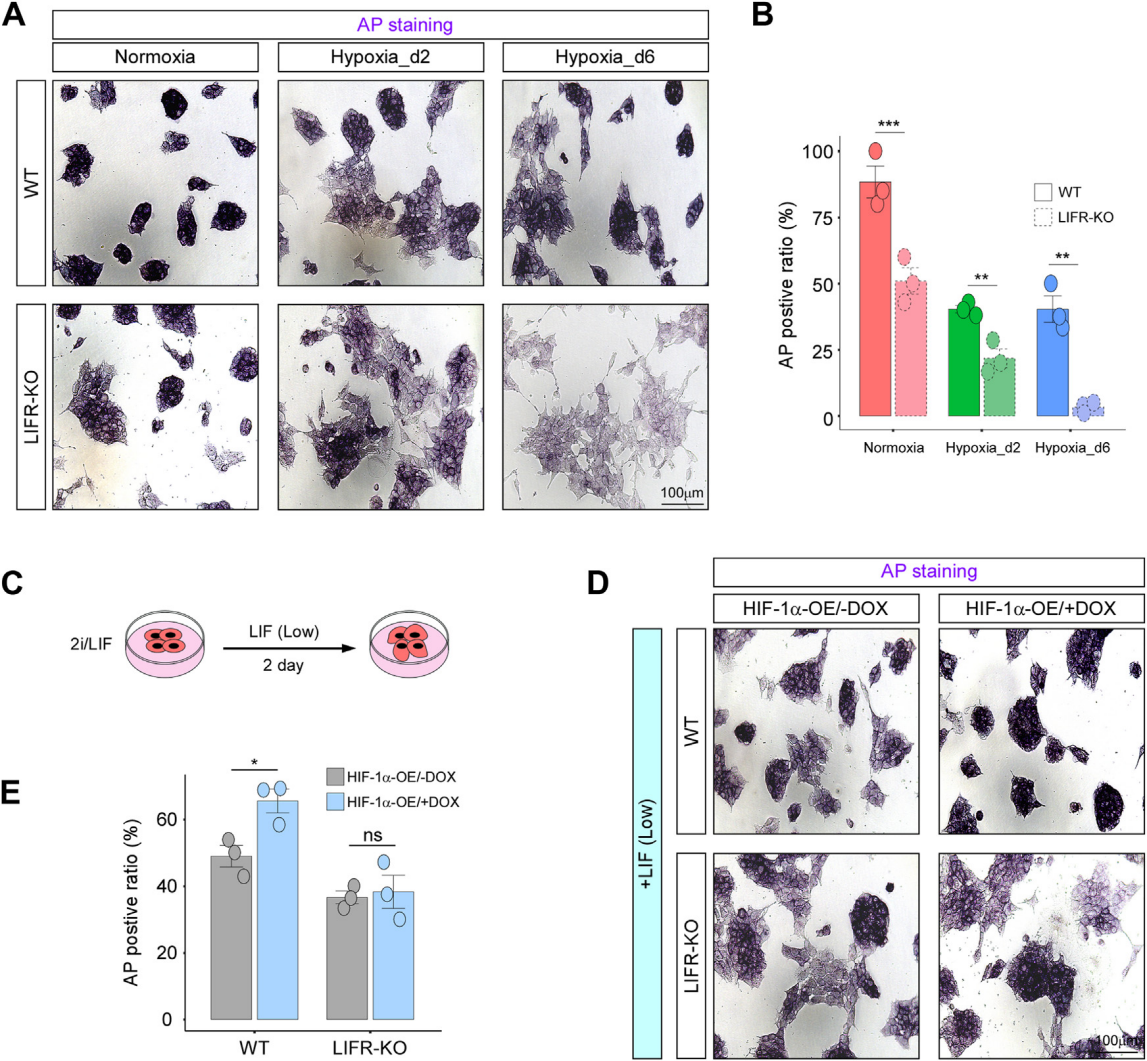
**LIFR mediated the function of HIF-1α in regulating mESC pluripotency during oxygen transition.***A*, AP staining of wildtype and LIFR knockout ESCs was performed under normoxic conditions (Normoxia), hypoxia for 2 days (Hypoxia_d2), and hypoxia for 6 days (Hypoxia_d6). *B*, quantification of positive cell colonies. n = 3. *C*, a schematic diagram illustrating the culture of cells under normoxic conditions, transitioning from 2i/LIF to a low-dose LIF for 2 days. *D*, AP staining of wildtype and LIFR knockout cells in HIF-1a overexpression and (*E*) quantification of their positive ratio. n = 3. ∗*p* < 0.05; ∗∗*p* < 0.01; ∗∗∗*p* < 0.001; ns, not significant. AP, alkaline phosphatase; HIF-1α, hypoxia-inducible factor 1-alpha; LIF, leukemia inhibitory factor; mESC, mouse embryonic stem cell.

To investigate the molecular mechanism by which HIF-1α regulates the pluripotency of mESCs, we hypothesized that LIFR acts as a downstream effector of HIF-1α, based on prior HIF-1α ChIP-Seq and HIF-1α knockdown RNA-Seq data (Fig. 5, *E* and *F*). As indicated by AP staining, LIFR knockout resulted in more pronounced differentiation of stem cells compared with wildtype under normoxic, acute hypoxic, and stable hypoxic conditions (Fig. 7, *A* and *B*). These findings suggested that LIFR is integral to the maintenance of stem cell pluripotency.

To further investigate the interplay between HIF-1α and LIFR in the regulation of stem cell pluripotency, we performed LIFR knockout in HIF-1α-overexpressing cells. AP staining revealed that HIF-1α overexpression in wildtype cells significantly preserved stem cell pluripotency after 2 days of culture in the presence of a low dose of LIF. However, this effect was completely abolished in LIFR knockout cells, indicating that HIF-1α sustains pluripotency primarily through LIFR (Fig. 7, *C*–*E*).

**Figure 8:**
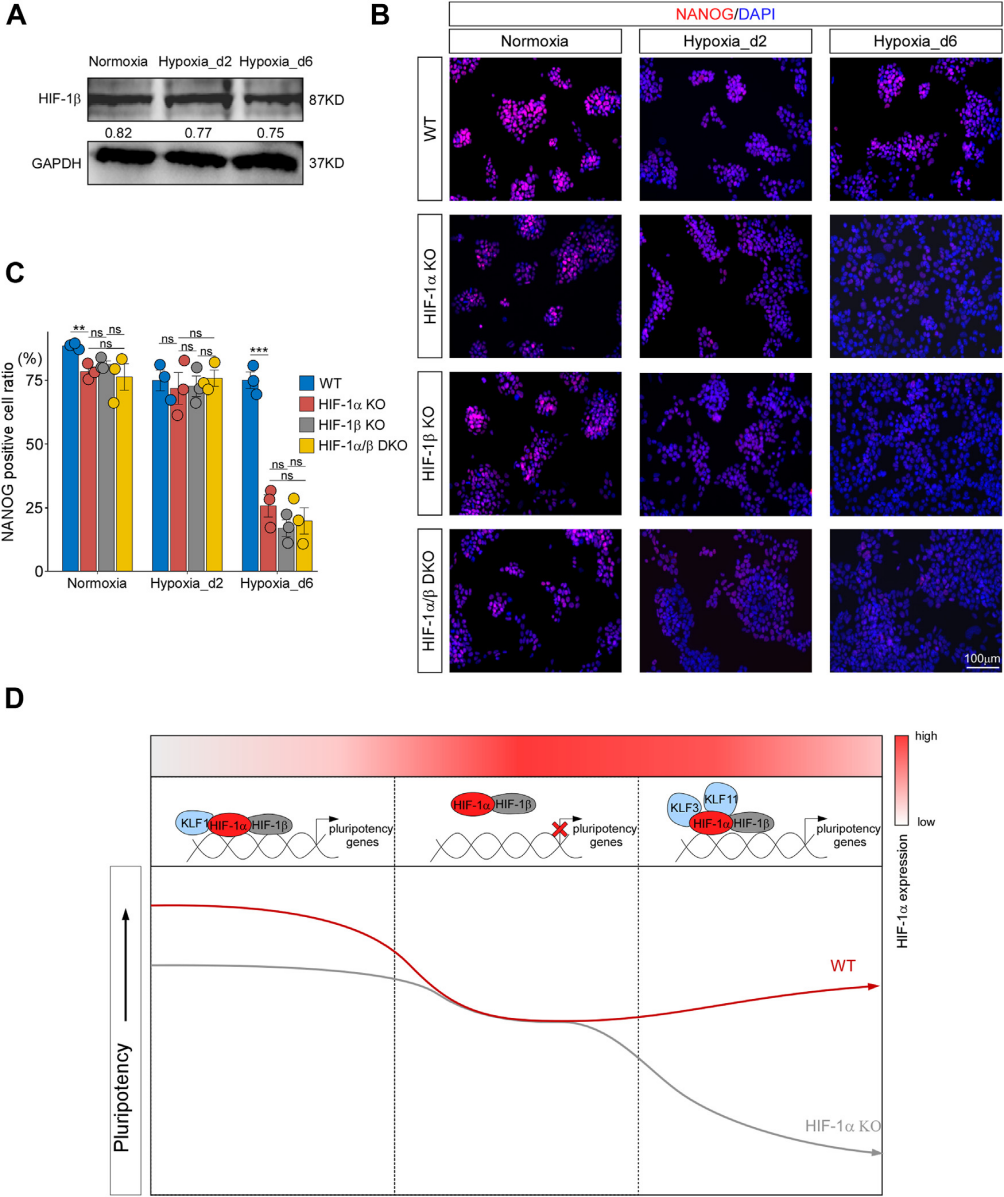
**HIF-1β was stably expressed and cooperated with HIF-1α in regulating mESC pluripotency during oxygen transition.***A*, HIF-1β protein expression and its quantitative analysis in the Normoxia, Hypoxia_d2, and Hypoxia_d6 groups. n = 3. *B* and *C*, NANOG immunofluorescence staining of wildtype (WT), HIF-1α knockout (HIF-1α KO), HIF1β knockout (HIF-1 KO), and HIF1α/HIF1β double knockout (HIF-1α/β DKO) ESCs across the three conditions. *D*, a working model of HIF-1α in regulating mESC pluripotency during oxygen transition. n = 3. ∗∗*p* < 0.01; ∗∗∗*p* < 0.001; ns, not significant. HIF-1α, hypoxia-inducible factor 1-alpha; mESC, mouse embryonic stem cell.

HIF-1α must dimerize with the HIF-1β subunit to function as a TF (24). To explore the role of HIF-1β in response to oxygen fluctuations, we conducted Western blot analysis, which showed no significant change in the expression of HIF-1β under Normoxia, Hypoxia_d2, and Hypoxia_d6 (Fig. 8*A*). Furthermore, NANOG immunofluorescence staining results demonstrated that HIF-1β knockout in all three conditions led to similar outcomes as HIF-1α knockout. Specifically, HIF-1β was crucial for maintaining mESC pluripotency under stable normoxia and hypoxia but not under acute hypoxia. Notably, double knockout of HIF-1α and HIF-1β did not yield any additional effects compared with the single knockouts, suggesting that HIF-1α, acting as an oxygen-sensitive factor, collaborates with HIF-1β to maintain stem cell pluripotency during oxygen transitions (Fig. 8, *B* and *C*).

Given that hypoxia treatment significantly impaired mESC pluripotency, HIF-1α not only helped maintain pluripotency but also prevented further loss of pluripotency under stable hypoxia. According to our motif analyses, HIF-1α cooperated with different TFs in the Normoxia, Hypoxia_d2, and Hypoxia_d6 groups (Fig. 2, *C* and *D*). This suggested that the biased function of HIF-1α during the transition from normoxia to hypoxia might have been linked to different cooperating TFs, warranting further investigation (Fig. 8*D*).

## Discussion

HIF-1α was a crucial TF that mediates responses to hypoxia and, as recent reports suggested, can also function under normoxic conditions (25). However, the dynamics of HIF-1α target genes and their biological functions during the transition between normoxia and hypoxia have not been thoroughly explored. In this study, we discovered that HIF-1α exhibits a "bind–release–bind" pattern when transcriptionally regulating target genes during the normoxia-to-hypoxia transition. Specifically, HIF-1α regulated mESC pluripotency–related genes under both stable normoxia and hypoxia but not during the intermediate stage of the transition. Consequently, the loss of HIF-1α significantly impaired mESC pluripotency under stable normoxia and hypoxia but not at the intermediate stage. This biased binding and function of HIF-1α appeared to be partially because of its interaction with different cooperating TFs under varying conditions.

Hypoxia and HIF-1α are essential in regulating ESC pluripotency and differentiation, but findings in this area have been conflicting. Some studies have suggested that hypoxia and HIF-1α synergistically promote ESC pluripotency and inhibit differentiation (25, 26, 27), whereas others have indicated that hypoxia facilitates differentiation and impairs pluripotency (17, 28, 29). Moreover, the role of HIF-1α in ESC pluripotency and differentiation is highly variable. In our study, we found that wildtype mESC pluripotency was well maintained under normoxia but significantly impaired under both acute and stable hypoxia, with both conditions having equivalent impacts. HIF-1α maintained mESC pluripotency under stable normoxia and hypoxia but not during acute hypoxia—the intermediate transition stage—aligning with its binding pattern to pluripotency-related genes. Notably, HIF-1α expression peaks at the intermediate stage, indicating that its lack of regulation of pluripotency-related genes during this phase is not because of a deficiency of HIF-1α itself. These results suggested that different atmospheric conditions and HIF-1α levels jointly influenced mESC pluripotency, potentially explaining the conflicting results in previous studies.

HIF-1α governs gene expression changes in response to oxygen levels. Despite extensive research on HIF-1α target genes across various cell types, microenvironments, and cell states (30, 31), a comprehensive profile of HIF-1α binding dynamics during the normoxia-to-hypoxia transition has been lacking. Our Western blot analyses showed that HIF-1α is expressed throughout the transition, with peak expression at the intermediate stage, corroborating previous reports (32, 33). However, ChIP-Seq analysis revealed that HIF-1α binds to the fewest peaks and target genes during the transition stage, despite its abundance. GO analysis indicated that HIF-1α target genes were enriched for mESC pluripotency maintenance under stable normoxia and hypoxia but not during the transition stage, suggesting biased regulation by HIF-1α based on atmospheric conditions. This finding was confirmed by functional assays, including AP staining, OCT4 immunostaining, and RT–quantitative CR of pluripotency markers. Further analysis showed that the g5 gene set, which included genes targeted by HIF-1α under stable normoxia and hypoxia but not during the transition, was enriched for mESC pluripotency. Integrative analysis of ChIP-Seq and RNA-Seq data identified Lifr as a key gene that mediated the biased functions of HIF-1α under different conditions. The JAK–STAT3 pathway, crucial for LIFR-mediated mESC pluripotency, was enriched in HIF-1α targets under stable conditions but not during the transition. The effect of hypoxia and HIF-1α on the JAK–STAT pathway has been debated. Some reports have suggested that HIF-1α inhibits the LIFR–STAT3 pathway under hypoxia (17, 34), whereas other studies have indicated that hypoxia has no effect on STAT3 activity in mESCs (35). Our results indicated that HIF-1α regulated mESC pluripotency *via* the LIFR-mediated JAK–STAT pathway, warranting further investigation.

In addition to the regulation of stem cell pluripotency, g5 genes also include some genes related to cell fate determination and cell differentiation, such as *Fam183b* and *Ndrg1*. Previous studies have reported that *Fam183b* is expressed in the left–right organizer region of early mouse embryos (36), where HIF-1α expression occurs independently of hypoxia (37). Functionally, consistent with *Fam183b*, normoxic HIF-1α is crucial for ciliogenesis (38). Combined with HIF-1α ChIP data, these findings suggest that HIF-1α may regulate the expression of *Fam183b* in a stage-specific manner during oxygen transitions.

NDRG1 is a member of the N-myc downregulated gene (NDRG) family and is repressed in a Myc-dependent manner (39). MYC plays an important role in maintaining stem cell self-renewal and pluripotency, and naive ground state ESCs usually contain low levels of MYC (40). In addition, it has been reported that *Ndrg1* is directly or indirectly regulated by HIF-1α in an oxygen-dependent manner, thereby affecting cancer metabolism and tumor progression (41). Based on the role of HIF-1α in stem cell pluripotency, we hypothesized that NDRG1 plays an important role in the maintenance of stem cell pluripotency. However, this needs to be confirmed by further experiments.

Given the continuous expression of HIF-1α during the transition from normoxia to hypoxia, we investigated whether certain genes require constant HIF-1α regulation. Analysis of shared HIF-1α target genes under stable normoxia, hypoxia, and the intermediate transition stage (termed the g7 gene set) revealed associations with cell fate commitment, stem cell differentiation, translation, and response to extracellular stimuli. The g7 genes were enriched in pathways, such as pyruvate metabolism, mitogen-activated protein kinase, HIF-1, PI3K–Akt, and transforming growth factor-beta signaling, indicating that key metabolic processes and pathways were under constant regulation by HIF-1α. Classical HIF-1α targets, such as *Ldha* and *Pgk1*, are directly regulated by HIF-1α and play crucial roles in cellular metabolism and glycolysis, processes that are essential for cellular response and adaptation to hypoxic environments (42, 43). These genes are continuously regulated by HIF-1α during oxygen transitions, not only confirming previous reports on HIF-1α target genes but also ensuring the survival benefits of HIF-1α-mediated staged regulation. Notably, the binding strength of HIF-1α to these genes varied, suggesting that further studies are needed to determine if this affects gene expression and pathway activities.

Finally, we explored the mechanisms underlying the dynamic binding behavior of HIF-1α. TF binding can be influenced by cofactors, chromatin status, and histone modifications. Motif analysis using the HOMER algorithm identified TF cofactors that interact with HIF-1α under different atmospheric conditions, potentially explaining the biased HIF-1α binding. However, we cannot rule out the impact of chromatin status and histone modifications. Hypoxia and HIF-1α have been reported to increase genome-wide bivalent epigenetic marking in cellular reprogramming of muscle stem cells and to associate with histone H3K4me3 modifications (44, 45, 46). HIF-1α also plays a role in maintaining histone methylation homeostasis, as it targets JmjC-containing histone demethylases in HepG2 and U87 cells (47). In addition, HIF-1α recruits cyclin-dependent kinase 8 to stimulate RNAPII elongation in colorectal cancer (48). Future integrative studies of chromatin status, histone modifications, and HIF-1α binding are needed to better understand these mechanisms.

In conclusion, HIF-1α exhibited a "bind–release–bind" pattern toward its target genes, especially those involved in mESC pluripotency maintenance, during the normoxia-to-hypoxia transition. As a result, HIF-1α regulated mESC pluripotency under stable normoxia and hypoxia but not during the intermediate transition stage. This biased binding profile was not because of HIF-1α expression levels but was partially attributed to its cooperating TFs. Our study unveiled the dynamics of HIF-1α target genes and their roles in mESC pluripotency, shedding light on the controversies surrounding hypoxia and HIF-1α′s roles in mESC pluripotency, and enhancing our understanding of the HIF-1α working model under different atmospheric conditions.

## Experimental procedures

### Cell culture

The AB2.2 mESCs were obtained from the Stem Cell Bank of the Chinese Academy of Sciences. These cells were authenticated by short tandem repeat profiling and confirmed to be free of mycoplasma contamination. The AB2.2 mESCs were cultured in the KnockOut Dulbecco's modified Eagle's medium (Gibco) supplemented with 15% fetal bovine serum (ExCell), 50 U/ml penicillin (Gibco), and 50 μg/ml streptomycin (Gibco), 2 mM l-glutamine (Gibco), 1× MEM Nonessential Amino Acids Solution (Gibco), and 0.1 mM 2-mercaptoethanol (Sigma). To maintain stem cell pluripotency, the medium was also supplemented with 3 μM CHIR99021 (MCE), 1 μM PD0325901 (MCE), and 103 units LIF (Millipore). For low doses of LIF, 100 units are often used. Cell culture dishes for AB2.2 mESCs were coated with 0.1% gelatin (Sigma). Cell culture under normoxia was conducted by incubating cells in a humidified incubator (Thermo Fisher) with 5% CO_2_ at 37 °C, whereas cell culture under hypoxia was achieved by incubating cells in a trigas incubator (Thermo Fisher) with 2% O_2_ and 5% CO_2_ at 37 °C. All cell lines used in this study were routinely tested for mycoplasma contamination using a Mycoplasma qPCR Detection Kit (Beyotime) according to the manufacturer's instructions.

### Plasmids and cell lines

HIF-1α knockdown and scramble control plasmids were constructed by ligating the designed HIF-1α and scramble shRNA fragments into the pLL4.0 vector at the HpaI and XhoI enzyme sites, respectively. The pcDNA3 mHIF-1α MYC (P402A/P577A/N813A) plasmid, which encodes an oxygen-resistant variant of HIF-1α, was obtained from Addgene. The gene fragment encoding oxygen-resistant HIF-1α was subsequently ligated into the pCW57-MCS1-2A-MCS2 plasmid, generating a doxycycline-inducible expression plasmid for oxygen-resistant HIF-1α.

These plasmids were cotransfected with the psPAX2 and pMD2.G plasmids using the PolyJet (SignaGen) into 293T/17 cells to produce corresponding lentiviruses, respectively. AB2.2 mESCs were infected with lentiviruses in the presence of polybrene and selected with 2 μg/ml puromycin until stable to generate shHIF-1α and HIF-1α-OE mESCs. Our previous studies have reported HIF-1α knockdown and overexpression in shHIF-1α and HIF-1α-OE mESCs using Western blot analysis (33).

Knockout ESCs were established as previously described (37). The HIF-1α, HIF-1β, and LIFR knockout plasmids were generated by ligating the corresponding guide RNA fragments into the lentiCRISPRv2 vector. The resulting plasmids were transfected into cells using Lipofectamine 3000 (Invitrogen) according to the manufacturer’s instructions. Drug selection was initiated 48 h post-transfection. Knockout cell lines were then generated through single-cell isolation and cloning, followed by PCR genotyping for confirmation. The primer sequences are listed in Table S1.

### ChIP-Seq and data analysis

ChIP-Seq experiments targeting H3K4me3 and H3K27ac were performed on mESCs cultured under normoxia and 2-day hypoxia, respectively. All immunoprecipitation procedures were executed using the ChIP-Seq High Sensitivity Kit (Abcam) in strict accordance with the manufacturer's protocol. Library construction and sequencing were performed on the Illumina NovaSeq X Plus by the Novogene Co, Ltd. ChIP-Seq data related to HIF-1α in mESCs were downloaded from the Gene Expression Omnibus (GEO) database with the accession number GSE195545. The data were filtered by the trim_galore (v0.6.7) and aligned to the mm10 mouse genome using the bowtie2 (v2.3.5.1). The aligned reads were then sorted and indexed using the samtools (v1.7). PCR duplicates were removed using the samtools markup function. The biological replicates of each group were merged using the samtools merge function. All aligned bam files were visualized using the IGV (v2.9.4) software.

ChIP-Seq peaks were determined using the MACS2 (v2.2.7.1) with the default parameters. The input ChIP-Seq was used as the control. Peaks were annotated with the ChIPseeker (v1.30.3) based on the mm10 mouse genome in R (v4.1.0). Target genes of specific ChIP peaks were defined as the genes whose TSSs were the nearest to the peaks and the distances were less than 3 kb. The distributions of ChIP peaks on different regions of gene locus were analyzed using the plotAnnoBar function in the ChIPseeker. The distributions of the distances of ChIP peaks to the TSSs were analyzed using the plotDistToTSS in the ChIPseeker.

Bam files were converted to bigWig files using the bamCoverage function in the Deeptools (v3.5.1) by setting the parameters as “-bs 1 --normalizeUsing CPM.” The bigWig files were then analyzed by the computeMatrix reference-point function in the Deeptools. The signals within TSS ± 3 kb regions were included in the calculations. ChIP-Seq peak heatmaps and mean intensity curves were plotted with the plotHeatmap and plotProfile functions in the Deeptools, respectively. The narrowPeak files derived from MACS2 callpeak were analyzed with the findMotifsGenome function in the HOMER (v4.11) against the mm10 mouse genome using the default parameters.

### RNA-Seq and data analysis

The total RNA of the scramble control (Ctrl) and HIF-1α knockdown (shHIF) mESC cells that were stably cultured under normoxia or treated with 2-day acute hypoxia was extracted using a total RNA isolation reagent (Biosharp). The total RNA samples were sent to the Novogene Co Ltd for library construction and RNA-Seq. The raw data were first trimmed and filtered using the trim_galore (v0.6.7) and aligned to the mm10 mouse genome using the HISAT2 (v2.2.1). Read counts were statistically generated using the StringTie (v2.2.1). Principal component analysis was conducted using the FactoMineR (v2.8) package in R. DEGs were determined by the DESeq2 (v1.34.0) in R. The fold change of gene expressions >1.5 and adjusted *p* value <0.05 were used as the cutoff criteria. Gene expression heatmap was plotted using the pheatmap (v1.0.12) package in R.

### GO and KEGG pathway analyses

Both GO and KEGG analyses were performed using the compareCluster function in the clusterProfiler (v4.2.2) R package. For GO analysis, the parameters were set as follows: fun = "enrichGO," OrgDb = "org.Mm.eg.db," ont = "BP," and pvalueCutoff = 0.05, pAdjustMethod = "BH." For KEGG analysis, the parameters were set as follows: fun = "enrichKEGG," organism = "mmu," and pvalueCutoff = 0.05. The dotplots displaying the GO and KEGG analysis results were produced using the ggplot2 (v3.4.2) in R.

### Western blot analysis

Protein extraction and Western blotting were performed as previously described. Briefly, the cells were lysed on ice using Cell Lysis Buffer (Beyotime) containing EASYpack protease inhibitors (Roche). Protein concentration was determined using the BCA assay (Biosharp), and the equal concentration of samples was then loaded onto a 10% SDS-PAGE gel for electrophoretic separation. Subsequently, the proteins were transferred onto polyvinylidene fluoride membranes. The membranes were blocked with 5% Tris-buffered saline with Tween-20 milk and incubated overnight with primary antibody. The next day, the membranes were incubated with donkey anti-rabbit horseradish peroxidase secondary antibody (1:2000 dilution; Invitrogen; catalog no.: 31458) and treated with ECL chemiluminescence substrate (Biosharp) to produce signals, which were captured using the Tanon 5200 Imaging System (Tanon). Gel band intensities were quantified using ImageJ (Fiji) software. The primary antibodies were used: anti-HIF1α pAb (1:100 dilution; Novus, catalog no.: NB100-479) and anti-HIF1β pAb (1:1000 dilution; ABclonal, catalog no.: A0972).

### AP staining

AP activity was detected using the BCIP/NBT Alkaline Phosphatase Color Development Kit (Beyotime) following the manufacturer’s protocol. Briefly, mESCs were seeded on gelatin-coated 6-well plates at a density of 1000 cells per well and cultured for 6 days before AP staining. Subsequently, the cells were fixed with 4% paraformaldehyde for 10 min and washed twice with TBS buffer. Finally, the cells were stained for 30 min at room temperature and washed briefly with ddH2O. Staining outcomes were observed using a Leica DMi8 microscope.

### Immunofluorescence assay

The ESCs were fixed with 4% paraformaldehyde for 10 min and then blocked with 10% normal goat serum and 0.1% Triton X-100 in PBS. The cells were incubated overnight with primary antibody. On the next day, the cells were incubated with secondary antibodies for 90 min, followed by 4',6-diamidino-2-phenylindole staining for 5 min. Images were taken with a Leica DMi8 fluorescence microscope. The following antibodies were used: anti-OCT4 monoclonal antibody (1:100 dilution; Santa Cruz, catalog no.: sc-5279), anti-NANOG monoclonal antibody (1:100 dilution; Cell Signaling, catalog no.: 8822S), goat anti-mouse Alexa Fluor Plus 555-conjugated IgG (1:500 dilution; Invitrogen, catalog no.: A32727), and goat Anti-rabbit IgG H&L Alexa Fluor 555-conjugated IgG (1:500 dilution; Abcam, catalog no.: ab150078).

### Real time-quantitative PCR

Total RNA was extracted using a total RNA isolation reagent (Biosharp), followed by reverse transcription using the FastKing RT kit (Tiangen). Quantitative PCR was performed with the PowerUp SYBR Master Mix (Applied Biosystems). All procedures were carried out according to the respective manufacturer’s instructions. β-actin was used as an internal control. The sequences of the RT–quantitative PCR primers are listed in Table S2.

### Lactate measurement

Lactate levels were assessed using a commercial lactate (LD) assay kit (Nanjing Jiancheng Bioengineering Institute) according to the manufacturer’s instructions. Briefly, cells were lysed in PBS by freeze–thaw cycles, and the supernatants were collected after centrifugation at 4000*g* for 10 min at 4 °C. The reaction mixture was prepared in 1.5 ml microcentrifuge tubes by adding lactate chromogenic and enzyme reagents, followed by incubation at 37 °C for 10 min. Absorbance was measured at 530 nm using a microplate reader.

### Statistical analysis

All statistical analyses were performed in R. All experiments performed at least three independent replications or technical replications, as indicated in the figure legends. Statistical significance was determined using Student’s *t* test for two-group comparisons and one-way ANOVA for three or more group comparisons. *p* < 0.05 was the threshold of significance. All data were presented as mean ± SD.

## Data availability

The data generated in this study are available from the corresponding author upon reasonable request. The bulk RNA-Seq and ChIP-Seq data generated in this study have been deposited in the GEO database under accession numbers GSE268827 and GSE294812, respectively. The ChIP-Seq data related to HIF-1α in mESCs were retrieved from the GEO using the accession number GSE195545.

## Supporting information

This article contains supporting information.

## Conflict of interest

The authors declare that they have no conflicts of interest with the contents of this article.
