# Supplementary material for: The stage-specific roles of HIF-1α in regulating mESC pluripotency during oxygen transition
Source: J Biol Chem. 2025 Jun 6;301(7):110344. doi: 10.1016/j.jbc.2025.110344 (PMC12269511; doi:10.1016/j.jbc.2025.110344)
Supplement: Supporting Information Figures [file mmc1.docx]

**
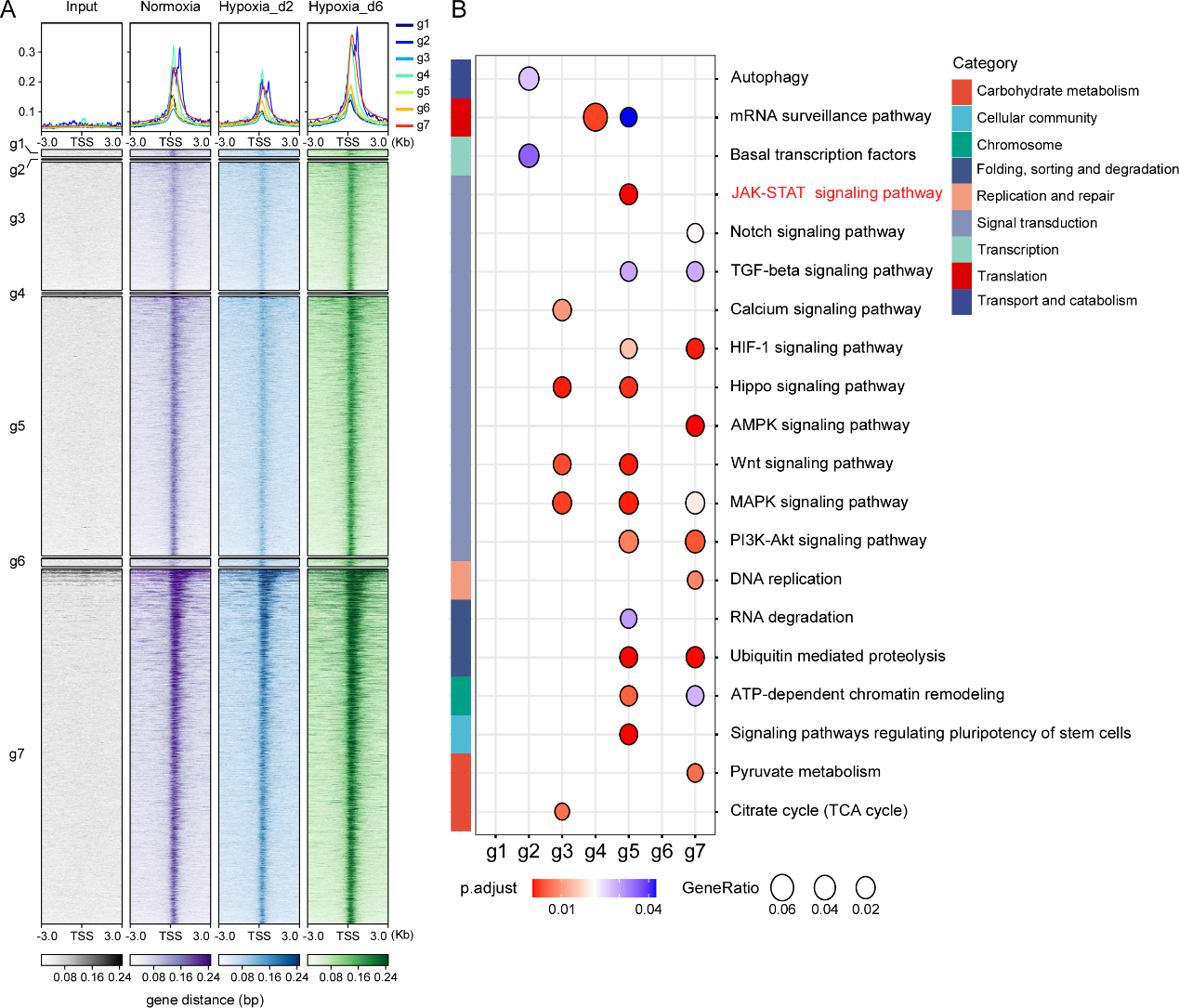
Figure S1. The g1~g7 gene sets targeted by HIF-1α during the transition from normoxia to hypoxia.** (A) The heatmaps and intensity plots of HIF-1α ChIP-seq at the seven gene sets. (B) The KEGG pathway analysis on the seven gene sets.

**
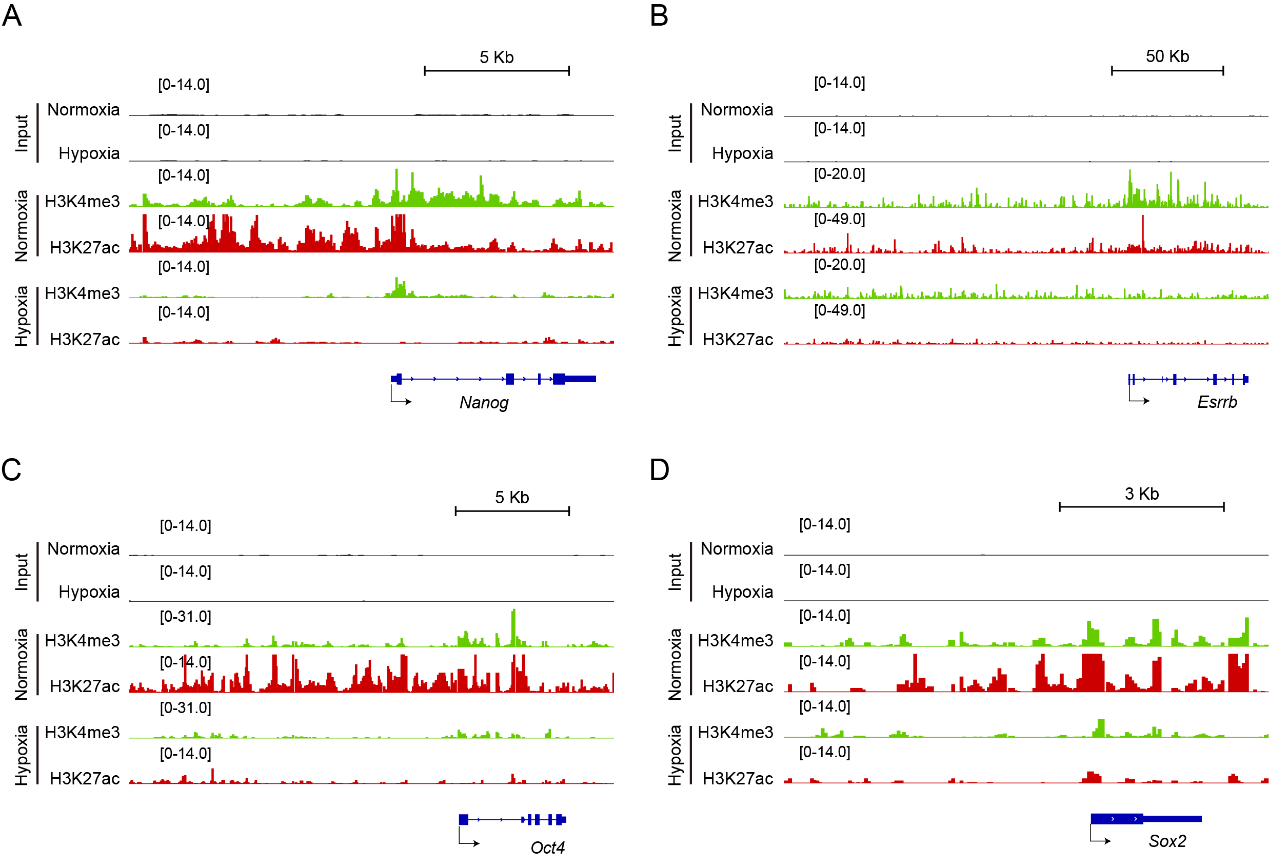
**

**Figure S2. ChIP-seq analysis of active histone modifications at pluripotency gene loci under normoxia and acute 2-day hypoxia.** ChIP-seq profiles of H3K4me3 and H3K27ac at the loci of (A) *Nanog*, (B) *Esrrb*, (C) *Oct4*, and (D) *Sox2* under normoxia and 2-day hypoxia.
