# Supplementary material for: The stage-specific roles of HIF-1α in regulating mESC pluripotency during oxygen transition
Source: J Biol Chem. 2025 Jun 6;301(7):110344. doi: 10.1016/j.jbc.2025.110344 (PMC12269511; doi:10.1016/j.jbc.2025.110344)
Supplement: Table S1 [file mmc2.docx]

**Table S1. List of PCR primers used for this study**

| **Primers** | **Sequences (5’-3’)** |
| --- | --- |
| shscramble-s | TGGGTGAACTCACGTCAGAATTCAAGAGATTCTGACGTGAGTTCACCCTTTTTTC |
| shscramble-as | TCGAGAAAAAAGGGTGAACTCACGTCAGAATCTCTTGAATTCTGACGTGAGTTCACCCA |
| ShHIF-1α-s | TGCTGTTGATCTTATAATGATTCAAGAGATCATTATAAGATCAACAGCTTTTTTC |
| ShHIF-1α-as | TCGAGAAAAAAGCTGTTGATCTTATAATGATCTCTTGAATCATTATAAGATCAACAGCA |
| KO-HIF-1α-F | CACCTGAACATCAAGTCAGCAACG |
| KO-HIF-1α-R | AAACCGTTGCTGACTTGATGTTCA |
| KO-HIF-1β-F | CACCGGGCTATTAAGCGACGGTCA |
| KO-HIF-1β-R | AAACTGACCGTCGCTTAATAGCCC |
| KO-LIFR-F | CACCGCGACGATGTGTACGGAACGG |
| KO-LIFR-R | AAACCCGTTCCGTACACATCGTCGC |
