# Supplementary material for: The stage-specific roles of HIF-1α in regulating mESC pluripotency during oxygen transition
Source: J Biol Chem. 2025 Jun 6;301(7):110344. doi: 10.1016/j.jbc.2025.110344 (PMC12269511; doi:10.1016/j.jbc.2025.110344)
Supplement: Table S2 [file mmc3.docx]

**Table S2. List of RT-qPCR primers used for this study**

| **Primers** | **Sequences (5’-3’)** |
| --- | --- |
| *Nanog*-F | TCTTCCTGGTCCCCACAGTTT |
| *Nanog*-R | GCAAGAATAGTTCTCGGGATGAA |
| *Esrrb*-F | CCAGAACCATCCAGAATCTTC |
| *Esrrb*-R | AAACCCAGGCTACCTTCCAT |
| *Zfp42*-F | GCATCGCTGTGGGCATTAG |
| *Zfp42*-R | GTGAGGCGATCCTGCTTTCT |
